# Consensus‐based clinical guidelines for ambulatory electromyography and contingent electrical stimulation in sleep bruxism

**DOI:** 10.1111/joor.12876

**Published:** 2019-09-11

**Authors:** Frank Lobbezoo, Ghizlane Aarab, M. Oliver Ahlers, Lene Baad‐Hansen, Olaf Bernhardt, Eduardo E. Castrillon, Nikolaos Nikitas Giannakopoulos, Anders Grønbeck, Justus Hauschild, Marianne Holst‐Knudsen, Naja Skovlund, Magdalini Thymi, Peter Svensson

**Affiliations:** ^1^ Department of Orofacial Pain and Dysfunction Academic Centre for Dentistry Amsterdam (ACTA) University of Amsterdam and Vrije Universiteit Amsterdam Amsterdam The Netherlands; ^2^ CMD‐Center Hamburg‐Eppendorf Hamburg Germany; ^3^ Department of Prosthetic Dentistry School of Dental Medicine University Medical Centre Hamburg‐Eppendorf Hamburg Germany; ^4^ Section of Orofacial Pain and Jaw Function Department of Dentistry and Oral Health Aarhus University Aarhus Denmark; ^5^ Scandinavian Center for Orofacial Neurosciences Aarhus Denmark; ^6^ Department of Restorative Dentistry, Periodontology, Endodontology, Preventive Dentistry and Pediatric Dentistry University Medicine Greifswald Greifswald Germany; ^7^ Department of Prosthodontics Faculty of Medicine University of Würzburg Würzburg Germany; ^8^ Section of Prosthetic Dentistry Department of Dentistry and Oral Health Aarhus University Aarhus Denmark; ^9^ Private Practice Isernhagen Germany; ^10^ Private Practice Herlev Denmark; ^11^ Department of Dental Medicine Karolinska Institutet Huddinge Sweden

**Keywords:** assessment, clinical guideline, contingent electrical stimulation, electromyography, management, sleep bruxism

## Abstract

As yet, there are still no evidence‐based clinical diagnostic and management guidelines for ambulatory single‐channel EMG devices, like the BUTLER^®^ GrindCare^®^ (GrindCare), that are used in patients with sleep bruxism. Therefore, a consensus meeting was organised with GrindCare developers, researchers, and academic and non‐academic clinicians experienced with the use of ambulatory EMG devices. The aim of the meeting was to discuss and develop recommendations for clinical guidelines for GrindCare usage, based on the existing clinical and research experience of the consensus meeting's participants. As an important outcome of the consensus meeting, clinical guidelines were proposed in which an initial 2‐week baseline phase with the device in its inactive (non‐stimulus) mode for habituation and assessment of the number of jaw‐muscle activities is followed by a 4‐week active phase with contingent electrical stimuli suppressing the jaw‐muscle activities. As to avoid the commonly reported reduction in sensitivity to the stimuli, a 2‐week inactive phase is subsequently installed, followed by a repetition of active and inactive phases until a lasting reduction in the number of jaw‐muscle activities and/or associated complaints has been achieved. This proposal has the characteristics of a single‐patient clinical trial. From a research point of view, adoption of this approach by large numbers of GrindCare users creates a great opportunity to recruit relatively large numbers of study participants that follow the same protocol.

## 1 INTRODUCTION

**Figure 1:**
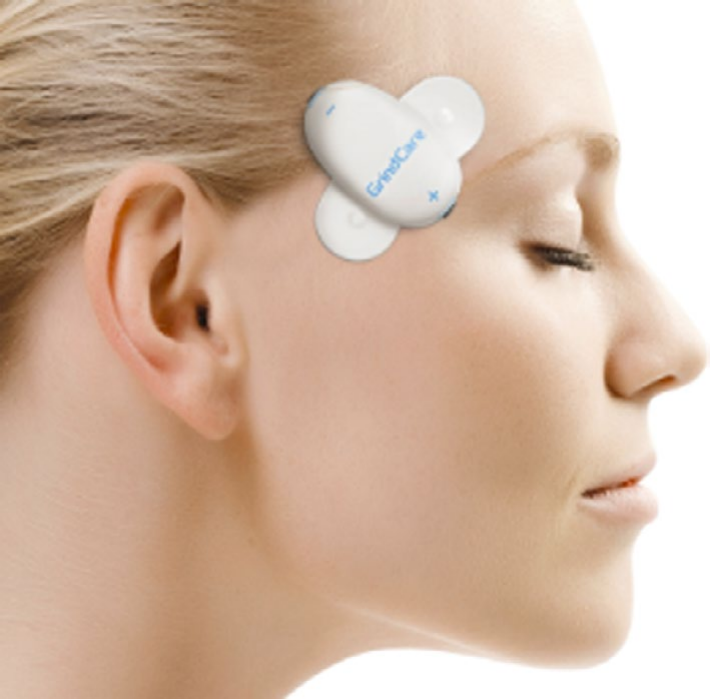
The BUTLER® GrindCare® sensor attached to the skin over the right temporal muscle. Courtesy of Sunstar Suisse S.A. [Colour figure can be viewed at wileyonlinelibrary.com]

Sleep bruxism has recently been defined as ‘a masticatory muscle activity during sleep that is characterised as rhythmic (phasic) or non‐rhythmic (tonic) and is not a movement disorder or a sleep disorder in otherwise healthy individuals.’^1^ From this definition, it can be gathered that sleep bruxism is not a disorder, but ‘just’ a jaw‐muscle activity that does not require extensive diagnostic procedures or management, unless it is associated with negative health outcomes, like severe temporomandibular disorder (TMD) pain, extensive tooth wear and/or dental restoration fractures/failures.^2^, ^3^, ^4^ In such cases, polysomnography (PSG; sleep recording) is considered the current gold standard diagnostic approach.^5^ While PSG is costly, time‐consuming, and has limited availability, a systematic review has identified several portable devices for the assessment of sleep bruxism, some of which yield promising accuracy.^6^ Amongst others, BUTLER^®^ GrindCare^®^ (GrindCare; Sunstar Suisse SA, Etoy, Switzerland; Figure 1) has been developed for the easy, wireless assessment of sleep bruxism in the home environment of the individual, based on single‐channel surface electromyography (EMG). The device is able to discriminate sleep bruxism diagnosed by PSG in a selected and otherwise healthy young population, and it may thus be a valid choice in clinical practice for the assessment of sleep bruxism.^7^ However, despite an increasing number of publications over the recent years,^8^, ^9^, ^10^, ^11^, ^12^, ^13^ there are as yet no evidence‐based clinical diagnostic guidelines for GrindCare or any other ambulatory single‐channel EMG devices.

As indicated above, when sleep bruxism is associated with one or more negative health outcomes, not only a valid assessment but also a safe and effective management is indicated. Unfortunately, the literature on the management of sleep bruxism has long been inconclusive due to the poor study designs employed.^14^ Happily, during the past decade, an increasing number of better‐quality studies on the management of sleep bruxism have been published.^15^ Nevertheless, there is still not enough evidence to define a standard of reference approach for the management of sleep bruxism. One of the more promising developments is the use of contingent electrical muscle stimulation, that is supposed to suppress the jaw‐closing muscle activity through trigeminal reflexes. This technique is the principle behind GrindCare, which may be a safe and effective management strategy for sleep bruxism. So far, however, this has only been studied in small‐scale research projects with highly selected study participants.^16^, ^17^, ^18^, ^19^, ^20^ As for the use of GrindCare in the diagnosis of sleep bruxism, there are still no evidence‐based clinical management guidelines for the device.

Since scientific evidence that could be used as the basis of clinical diagnostic and management guidelines is largely absent, a consensus meeting was organised with GrindCare developers, researchers, and academic and non‐academic clinicians experienced with the use of ambulatory EMG devices. The aim of the meeting was to discuss and develop recommendations for clinical guidelines for GrindCare usage, based on the existing clinical and research experience of the consensus meeting's participants.

## 2 METHODS

### 2.1 Participants

On 7 September 2018, a round‐table consensus meeting was organised at the Academic Centre for Dentistry Amsterdam (ACTA) with the ultimate aim to develop recommendations for consensus‐based clinical guidelines for BUTLER^®^ GrindCare^®^ (GrindCare) usage, based on consensus discussions among the attendants. In Table 1, the participants are briefly described. Prof. Dr Peter Svensson (Aarhus University, Aarhus, Denmark) and Dr Lorenz Uebersax (Sunstar Suisse SA, Etoy, Switzerland) were responsible for the selection procedure. As can be gathered from Table 1, a mixture of GrindCare developers (Sunstar Suisse SA, Etoy, Switzerland), bruxism researchers, and academic and non‐academic expert clinicians was invited. Three of the participants (FL, NNG and PS) are also members of the Academic Advisory Board for GrindCare, which is an unpaid role for two of them (FL and NNG).

**Table 1:** Participants^a^ of the round‐table consensus meeting at ACTA, Amsterdam, The Netherlands, on 7 September 2018: Name, Profession(s), Affiliation(s), role(s)

Name	Profession(s)	Affiliation(s)	Role(s)
Priv. Doz. Dr M. Oliver Ahlers^b^	Dentist, Specialist in Orofacial Pain and Dysfunction	Hamburg, Germany	GrindCare user, Expert clinician
Prof. Dr Olaf Bernhardt^b^	Dentist, Specialist and Professor in Orofacial Pain and Dysfunction	Greifswald, Germany	Grindcare user, Expert clinician, Bruxism researcher
Priv. Doz. Dr Nikolaos Nikitas Giannakopoulos^b^	Dentist, Specialist and Assoc. Professor in Prosthodontics (incl. Orofacial Pain and Dysfunction)	Würzburg, Germany	Member of the Academic Advisory Board for GrindCare, GrindCare user, Expert clinician, Bruxism researcher
Dr Anders Grønbeck^b^	Dentist, Specialist in Orofacial Pain and Dysfunction	Aarhus, Denmark	GrindCare user, Expert clinician
Dr Justus Hauschild^b^	Dentist, Specialist in Orofacial Pain and Dysfunction	Isernhagen, Germany	GrindCare user, Expert clinician
Dr Marianne Holst‐Knudsen^b^	Dentist, Specialist in Orofacial Pain and Dysfunction	Copenhagen, Denmark	GrindCare user, Expert clinician
Prof. Dr Frank Lobbezoo	Dentist, Specialist and Professor in Orofacial Pain and Dysfunction	Academic Centre for Dentistry Amsterdam (ACTA), The Netherlands	Member of the Academic Advisory Board for GrindCare, Expert clinician, Bruxism researcher
Naja Skovlund	Dental Assistant	Copenhagen, Denmark	GrindCare user
Prof. Dr Peter Svensson	Dentist, Specialist and Professor in Orofacial Pain and Dysfunction	Aarhus, Denmark	Moderator, Member of the Academic Advisory Board for GrindCare, Expert clinician, Bruxism researcher

a For Sunstar group, the following individuals were present as observers: Katharina Müller (Key Account Manager BUTLER^®^ GrindCare^®^, Sunstar Germany, Germany), Kamila Nieto (Product Manager, Sunstar Europe, Switzerland), Paola La Pietra (Clinical Affairs Manager, Sunstar Suisse, Switzerland), Dr Nao Takano (R&D Manager, Sunstar Suisse, Switzerland) and Dr Lorenz Uebersax (Clinical Affairs Director, Sunstar Suisse, Switzerland).

b Completed the questionnaire.

### 2.2 Questionnaire

Prior to the consensus meeting, the organisers (viz., Svensson and Uebersax) circulated a questionnaire amongst the participants, as to collect the participant's input on several selected GrindCare‐related topics1Please note that the questionnaire focused on the use of BUTLER® GrindCare® (Figure 1).:

- Functionality/reliability (ie 1. does the device measure jaw‐muscle activity and does it deliver contingent electrical stimuli and 2. do the GelPads provide sufficient attachment to the skin and/or do they cause skin irritation?);
- Function/validity (ie 1. does the device assess jaw‐muscle activity correctly and 2. does it apply the contingent stimuli correctly?);
- Patient benefit (ie do your patients benefit from 1. increased awareness of bruxism, 2. lifestyle adaptation, 3. the assessment of jaw‐muscle activities and/or 4. reduced jaw‐muscle activity?);
- GrindCare indication (ie is the device indicated for use in patients with 1. high bite forces while bruxing and/or 2. with many negative health outcomes?);
- Management protocol (ie 1. Is there an influence of differences in electrode positioning between nights, 2. is a stimulation‐free habituation phase needed, 3. after how much time should there be a positive effect of stimulation, 4. does the responsiveness to the stimuli reduce over time, 5. is there a need for interrupting the active phase, 6. should there be a maximum total management time and 7. can the device usage be combined with other management modalities?).

Depending on how the questions were formulated, they could either be answered as Yes, No or N/A, with or without a specification option. The responses to the questionnaire were used to get the round‐table discussion started. The discussion continued until full consensus was reached. The outcomes are elaborated below.

## 3 RESULTS

Six of the participants completed the questionnaire in advance. In Table 1, their names are indicated with a double asterisk. Below, the outcomes of the discussions are described per topic. Unless otherwise indicated, the reported issues are related to the BUTLER^®^ GrindCare^®^ (GrindCare; Figure 1).

### 3.1 Functionality/reliability

The discussion on the functionality/reliability topic mainly focused on possible detachment of the GelPads that has been observed and that could be a possible cause for unreliable functioning. Half of the respondents reported loss of stickiness, even after thorough skin cleansing. Consequently, the GrindCare device itself does not function properly, which was also reported in the questionnaire. Assuming that patients always apply fresh GelPads every time, the loss of stickiness is possibly, at least in part, skin temperature‐related or associated with a slight warming up of the device over time. As the stickiness cannot be further increased due to the concurrently increased risk of skin damage, the only available measure is to store the GelPads in a cool place prior to their application. In addition to the detachment issue, some cases of skin allergy have been encountered. This cannot be prevented other than by a thorough oral history and adequate user instructions.

### 3.2 Function/validity

The discussion on the function/validity topic zoomed in on the contradictory respondents' reports and published research findings. While only two of the respondents reported sufficient validity of the GrindCare in its assessment of jaw‐muscle activities when applied by the patients themselves at home, Stuginski‐Barbosa et al^7^ have demonstrated good concordance between electromyographically established jaw‐muscle activities by GrindCare version 3 (ie the fore‐last version of the GrindCare® [Medotech A/S, Herlev, Denmark] that existed of an electrode that was connected by a wire to a small EMG box attached to the body by means of a belt) and those established by polysomnography in a laboratory setting. If this finding can be extrapolated to the GrindCare remains to be studied, although the algorithm used by the GrindCare yields comparable results as a PSG‐based analysis of the same signal.^21^ Similarly, while only half of the respondents reported correct application of the contingent electrical stimuli, sufficiently high (but not painful) intensities of the stimuli were shown to be efficient in suppressing jaw‐muscle activities, which even resulted in significant decreases in jaw‐muscle symptoms like soreness, tiredness and unpleasantness.^20^ Possibly, the GrindCare users have not used sufficiently high stimulus intensities, which might, at least in part, explain the deviant clinical and scientific reports. Another cause could be the detachment of the GelPads (see above).

### 3.3 Patient benefit

All respondents agreed that patients increase their awareness of bruxism when using the GrindCare. Four out of six respondents reported that their patients benefit from the GrindCare in terms of lifestyle adaptation as well as of the assessment and reduction in jaw‐muscle activities. In the free text option, respondents indicated that their patients especially report reductions in oro‐facial pain, headache, neck pain, jaw tension, teeth sensitivity, disturbed sleep and hypertrophic jaw musculature. As per the discussion on the patient benefit topic, it should be noted that scientific evidence for such reductions is largely lacking. Recently, however, Shimada et al^20^ reported GrindCare‐related reductions in jaw‐muscle symptoms like soreness, tiredness and unpleasantness, but not in jaw‐muscle pain.

### 3.4 GrindCare indication

Especially, developing high bite forces while bruxing was frequently reported as an indication for GrindCare (4/6 respondents). To a much lesser extent, many negative health outcomes were considered an indication (2/6 respondents). While this latter outcome seems surprising, the discussion learned that all respondents were aware of the fact that scientific evidence for such indication is largely lacking and that at the same time all respondents expressed the desire to work as much as possible according to the principles of evidence‐based dentistry. It should be noted that the paper by Shimada et al^20^ was not available to the respondents at the time of the round‐table discussions.

### 3.5 Management protocol

The main part of the round‐table discussions dealt with the management protocol for GrindCare usage. In Table 2, the respective questions and the respondents' answers are summarised. The outcomes of the discussion on this topic are summarised below.

**Table 2:** Questions and answers of the respondents (n = 6) related to the management protocol topic of the questionnaire that was filled in before the round‐table consensus meeting, and the consensus that was reached during the meeting

Questions		Answers (number of Yes, No and N/A; and/or mean, median, and/or range of numeric answers) and consensus
1.	Is there an influence of differences in electrode positioning between nights?	Answers: Yes = 2, No = 2, N/A = 2 Consensus: Yes, but unimportant for measurement
2.	Is a stimulation‐free habituation phase needed? If Yes, how long?	Answers: Yes = 4, No = 1 and N/A = 1; mean = 7 d, median 7 d, range = 3‐14 d Consensus: 2‐wk baseline
3.	After how much time should there be a positive effect of stimulation?	Answers: Mean = 20 d, median 16 d, range = 2‐49 d Consensus: 4‐wk active phase
4.	Does the responsiveness to the stimuli reduce over time?	Answers: Yes = 3, No = 2, N/A = 1
5.	Is there a need for interrupting the active phase? If Yes, how long?	Answers: Yes = 3, No = 1, N/A = 2; range = 1‐12 wk Consensus: Dependent on the individual patient
6.	Should there be a maximum total management time? If Yes, how long?	Answers: Yes = 2, No = 3, N/A = 1; range = 2‐4 wk Consensus: No maximum
7.	Can the device usage be combined with other management modalities? If Yes, with which modalities?	Answers: Yes = 6, No = 0, N/A = 0 Consensus: Yes, viz., counselling, occlusal stabilization splint, medication, psychology

Regarding the first question of this topic, all participants to the discussion agree that while differences in the positioning of the GrindCare between nights influence the measurements, they seem unimportant for the diagnostic and management functions of the device.

**Figure 2:**
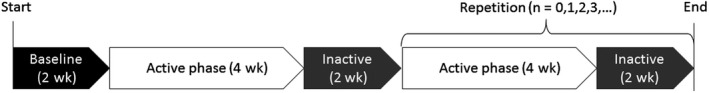
Schematic representation of the consensus‐based clinical guidelines for BUTLER® GrindCare® usage: timing of the diagnostic and management phases

Questions 2‐6 of this topic are related to the timing of the diagnostic and management phases of the GrindCare usage. Consensus on this important issue was reached as described in Table 3. For a schematic representation, see Figure 2. Apart from the proposed clinical guidelines, the participants agree that there is a large variation between individual patients as to when a positive effect of GrindCare can be noted. Importantly, it is even pointed out that associated complaints can already improve in the absence of a reduction in the number of jaw‐muscle activities, and vice versa.

**Table 3:** Consensus‐based clinical guidelines for BUTLER® GrindCare® usage: timing of the diagnostic and management phases

Phase	Number of weeks
Baseline phase (without contingent electrical stimulation; for habituation and assessment of the number of jaw‐muscle activities)	2
Active phase (with contingent electrical stimulation; to reduce the number of jaw‐muscle activities; the average number of jaw‐muscle activities in the last 2 wk of this phase should be lower than the activity measured at baseline)	4
Inactive phase (without contingent electrical stimulation; to avoid commonly reported reduction in sensitivity to stimuli)	2
Repetition of active and inactive phases until a lasting reduction in the number of jaw‐muscle activities and/or associated complaints has been achieved	4 resp. 2

As for question 7, consensus was reached on the fact that management of bruxism always needs to be tuned at the individual patient's needs. Combination strategies will be part of such approach; see Discussion. At least, all patients will receive counselling, apart from GrindCare and/or any other possible management strategy for sleep bruxism (eg occlusal stabilization splint, medication, psychology; for an overview, see Lobbezoo et al^14^).

## 4 DISCUSSION

This paper describes the procedure and outcomes of a round‐table consensus discussion on the use and usefulness of the BUTLER^®^ GrindCare^®^ (GrindCare) in the assessment and management of sleep bruxism. The GrindCare shows promising reliability and validity, although reliability can be jeopardised by the commonly observed detachment of the device due to loss of stickiness of the GelPads. At the same time, the validity of the GrindCare has so far only partly been tested against the current gold standard, viz., polysomnography.^21^ As for the clinical effectiveness, relatively little research has been performed. Shimada et al^20^ showed that relatively high levels of contingent electrical stimulation yielded reductions in jaw‐muscle soreness, tiredness and unpleasantness, while jaw‐muscle pain was not affected. Clearly, more research is needed to further clarify the diagnostic and management characteristics of the GrindCare.

The proposed consensus‐based clinical guidelines for the assessment and management of sleep bruxism by means of GrindCare have the design of a single‐patient clinical trial.^22^ This means that the entire procedure has built‐in phases, during which the efficacy of the management can be judged in comparison to baseline (or inactive) phases. From a research point of view, adoption of this approach creates a great opportunity to recruit relatively large numbers of study participants that follow the same protocol. Importantly, these study participants will originate from different centres and regions worldwide, which will increase the external validity of the outcomes of such study. Of course, this will require extra attention for alignment with current privacy regulations of participating centres and clinics, but the gain of all efforts will be more insight into the clinical efficacy of the GrindCare in reducing the number of jaw‐muscle activities as well as in improving complaints that are associated with sleep bruxism.

The participants agreed that the proposed clinical guidelines for the application of the GrindCare in the management of sleep bruxism patients will have to be integrated into the established approaches for diagnosis and management at each individual clinic. For example, in everyday clinical practice, GrindCare is usually not applied as a stand‐alone management option for individual sleep bruxism patients; rather, combinations or integration into existing approaches are usually being sought. In highly specialised clinics like the ones at the Department of Orofacial Pain and Dysfunction at ACTA and at the Section of Orofacial Pain and Jaw Function at Aarhus University, such patients also receive counselling (ie information about the condition and advices on how to contribute to decreasing the jaw‐muscle activities by one's own effort) and, in the presence of concomitantly associated complaints like extensive mechanical tooth wear, an occlusal stabilization splint. In cases where this approach fails, GrindCare is added to the already installed management options (ie counselling and/or splint). Only when all these attempts still yield unsatisfactory outcomes, pharmacological strategies are added to the management protocol. Clearly, at least at ACTA and Aarhus University, GrindCare is part of a larger management protocol, and more research is needed to establish the validity of this protocol. A large‐scale study with that aim will be started shortly. Of course, the proposed clinical guidelines as described in the current paper will be implemented in that study's protocol. Other centres and clinics are encouraged to do that as well.

In conclusion, the consensus meeting that was organised with GrindCare developers, researchers, and academic and non‐academic clinicians yielded recommendations for clinical guidelines for GrindCare usage, based on the clinical and research experience of the consensus meeting's participants.
